# A holistic approach in epidemics

**DOI:** 10.3389/fpubh.2023.1263293

**Published:** 2023-11-13

**Authors:** Ioannis Tsagkarliotis, Nikolaos P. Rachaniotis

**Affiliations:** Department of Industrial Management and Technology, University of Piraeus, Piraeus, Greece

**Keywords:** holistic approach, epidemics, conceptual modelling, non-medical interventions, public health

## Abstract

This paper explores the concept of a holistic approach in preventing and responding to epidemics. Epidemics are defined as the occurrence of an illness or health-related event exceeding normal expectations within a specific community or region. Holism emphasizes viewing systems as a whole rather than a collection of parts. In the context of epidemics, a holistic approach considers not only medical interventions but also social, economic, psychological and environmental factors that influence disease transmission and management. The impact of climate change on epidemic response, the understanding of the significance of animal health and agriculture, the consideration of art, culture and societal factors, the exploration of the use of technology and innovation, the addressing of limitations in resources and the provision of enhanced support for the mental and emotional well-being of individuals and affected communities, are parts of this holistic approach. By integrating them, innovative practices as well as cross-sectoral and interdisciplinary techniques can be employed. Such an approach has the potential to enhance epidemic prevention and response strategies, ultimately contributing to positive public health outcomes.

## 1. Introduction

“*Epidemic is the occurrence in a community or region of cases of an illness, specific health-related behavior, or other health-related events clearly in excess of normal expectancy. The community or region and the period in which the cases occur must be specified precisely. The number of cases indicating the presence of an epidemic varies according to the agent, size and type of population exposed, previous experience or lack of exposure to the disease and time and place of occurrence*” (1).

On the other hand, Holism is a theory formulated by the South African statesman, military leader and philosopher Jan Christian Smuts in 1926 (2), according to which any system (physical, biological, social) should be viewed as whole, not merely as a collection of parts. He mainly used the term to describe his complex philosophy regarding the organization of nature.

Holism can provide insights and recommendations for improving epidemic response efforts by adopting a more comprehensive and integrated approach that takes into account the various interconnected factors that contribute to epidemic spread and response.

The objective of this paper is to investigate and evaluate the potential benefits of using a holistic approach in responding to epidemics. This may include exploring how a holistic approach can contribute to:

- More effective and efficient distribution of medical supplies and resources.
- Improved communication and coordination among different stakeholders involved in epidemic response.
- Better understanding and consideration of social and environmental factors that impact epidemic spread and response.
- Increased focus on promoting community engagement and empowerment in epidemic response efforts.
- Enhanced support for the mental and emotional well-being of individuals and communities affected by epidemics.

## 2. Background and literature review

Epidemics management is a complex and challenging task that involves various aspects like protection, prevention and treatment. Thoughtful preparation and swift mobilization of healthcare professionals and medical supplies are essential in slowing down or halting epidemics spread (3). Epidemic response requires the integration of multiple disciplines, including epidemiology, social sciences, diplomacy, logistics and crisis management. To achieve the best possible outcomes in addressing epidemics, a more holistic approach should be adopted.

In epidemics, a holistic approach entails considering all aspects of the outbreak, including medical interventions as well as social, economic and psychological factors that influence the spread of the disease. This approach acknowledges that epidemics are not only public health crises but also have far-reaching consequences for individuals, families, communities and the society as a whole. Therefore, a holistic approach aims to address all dimensions of an epidemic, encompassing prevention, diagnosis, treatment and recovery, suggesting that a comprehensive understanding of the whole is necessary to fully understand the individual parts (4). While reductionist thinking focuses on analyzing phenomena by studying their individual components, holistic thinking recognizes the interconnectedness and interdependence of these components (5).

Holistic medical care addresses the overall health of individuals, encompassing their physical, mental and emotional well-being, while also considering social factors (6–9). Implementing a holistic approach in healthcare policy leads to a better understanding of patients’ needs for treatment and helps in accepting self-responsibility during a pandemic (10, 11). It also aids in comprehending the transmission and infection mechanisms of a virus as well as the multi-level functioning of the immune system (12) combined with the immense vaccination efforts undertaken, as for example was the case for COVID-19 (13).

Relevant research has highlighted the importance of multi-sectoral collaboration, community engagement and health system strengthening as key components of a holistic approach to epidemics. Case studies of countries that have successfully implemented a holistic approach in their epidemic response, such as Vietnam (14) and South Korea (15) during the COVID-19 pandemic provide valuable lessons. The aspects of a holistic approach in epidemics that have been studied include:

- Community engagement: engaging with local communities and involving them in the planning and implementation of epidemic response measures has been found to be an important aspect of the holistic approach. Research has shown that community involvement can lead to increased acceptance of interventions and better outcomes (16).
- Mental health: the mental health of individuals affected by an epidemic can have a significant impact on their overall well-being and ability to recover (17). The importance of providing mental health support during and after an epidemic (18), especially in order to allay public fear and perception and encouraging people to avail routine healthcare services, irrespective of the status of the patient (19), is paramount.
- Social determinants of health: the social determinants of health, such as poverty, access to healthcare and education, have a significant impact on the spread and impact of an epidemic. The holistic approach addresses these factors (20, 21).
- Environmental factors: the environment plays a role in the transmission of a disease, and the importance of addressing environmental factors such as sanitation and hygiene in epidemic response has been highlighted (22).
- International cooperation: the global nature of epidemics requires international cooperation and collaboration (23, 24).
- Healthcare infrastructure: the healthcare infrastructure, including hospitals and healthcare workers, plays a critical role in epidemic response. Research has explored the importance of preparedness and capacity-building in healthcare infrastructure to respond effectively to epidemics (25).

This paper’s contribution is the development of a conceptual model setting the framework for a holistic approach in epidemics response. It includes identifying key components, outlining the relationships between different factors and proposing a structure to identify gaps and areas for further investigation.

## 3. Holistic approach model

A holistic approach in the case of epidemics has to deal with both preventing and responding and should be characterized by a general and spherical thinking (26), not limited to trivial and common practices already applied, but rather use innovative ones combining cross-sectoral and multi-interdisciplinary techniques. The proposed conceptual framework model provides a structured overview of the key components and outcomes associated with a holistic approach in epidemic response. It highlights the importance of interdisciplinary collaboration, comprehensive understanding and assessment, prevention and preparedness, integrated interventions, community engagement and empowerment, as well as continuity and resilience. Its major parts include:

### 3.1. The impact of climate change on epidemic response

Climate change has a significant impact on epidemic response by altering the transmission and distribution of infectious diseases. Changes in temperature, rainfall patterns and extreme weather events can affect the breeding and survival of disease vectors such as mosquitoes and ticks, as well as the habitat of animals that can carry and transmit diseases.

For example, as temperatures increase, the geographic range of some disease vectors can expand, increasing the risk of their transmission in new areas. In addition, changes in rainfall patterns can create new breeding grounds for disease-carrying mosquitoes, leading to increased transmission of diseases such as dengue fever and Zika virus. Extreme weather events such as floods and hurricanes can also lead to the displacement of people and animals, increasing the risk of disease transmission.

Climate change’s impact on the spread of infectious diseases has already been studied in the cases of malaria, Lyme disease and West Nile virus (27). Therefore, it is important for epidemic response strategies to take into account the potential impact of climate change and incorporate measures to mitigate its effects. Some potential strategies under a holistic perspective include improving surveillance systems to detect changes in disease transmission patterns, implementing mosquito control programs and promoting public health messaging and education campaigns to increase awareness of disease risks and prevention measures. Additionally, the efforts to reduce greenhouse gas emissions and mitigate the impacts of climate change can also indirectly benefit epidemic response efforts by reducing the potential for disease transmission.

### 3.2. The role of animal health and agriculture in epidemic response

Animal health and agriculture play a significant role in epidemic response, particularly in zoonotic diseases that can be transmitted from animals to humans. The holistic approach to epidemic response acknowledges the importance of animal health and agriculture as part of the larger public health system.

One aspect of the role of animal health and agriculture in epidemic response is the need for effective disease surveillance systems in animal populations. By detecting outbreaks of diseases in animals, public health officials can take preventative measures to limit the spread of the disease to humans. This requires collaboration between public health officials and those involved in animal health and agriculture, such as veterinarians and farmers (28).

Another aspect is the importance of safe and healthy food production practices. In the case of zoonotic diseases, it is essential to ensure that food products from animals are safe for human consumption (29). This requires a focus on animal health and hygiene, as well as safe food handling practices.

Research has also explored the role of animal vaccination programs in epidemic response (30). By vaccinating animals against diseases that can be transmitted to humans, such as avian influenza, public health officials can limit the spread of the disease and reduce the risk of human outbreaks.

### 3.3. The impact of art, cultural, and societal factors on epidemic response

Cultural and societal factors can have a significant impact on epidemic response. Beliefs and practices related to traditional medicine, burial rites and social gatherings can influence the spread of disease and the effectiveness of interventions. In some cases, cultural beliefs and practices may be at odds with public health recommendations, which can lead to resistance or mistrust (31).

This approach involves considering the cultural context in which an epidemic occurs (32), and understanding how art and cultural practices can be leveraged to promote health and resilience among affected populations. In the context of epidemic response, arts and culture can be used to disseminate important public health messages, reduce stigma and discrimination (33, 34) and promote mental and emotional well-being among affected individuals and communities.

The holistic approach to the role of arts and culture in epidemic response recognizes the potential for creativity and innovation in promoting health and well-being during an epidemic and emphasizes the significance of collaborative efforts with artists and cultural practitioners to develop appropriate and effective public health interventions. This approach recognizes the value of leveraging arts and culture as powerful tools for promoting positive health outcomes (35) and fostering community engagement in epidemic response, providing opportunities for artists to share their work, as well as incorporating artistic and cultural practices into public health programs and policies.

Research has explored the impact of cultural and societal factors on epidemic response in various contexts. In the Ebola outbreak in West Africa, traditional dance and music were used to educate people about the disease and promote preventive behaviors (36). A study in Sierra Leone found that community engagement and dialogue were crucial for building trust and addressing cultural practices related to burial rites during the Ebola outbreak (37). Another study in Uganda found that involving traditional healers in the response to cholera outbreaks improved community acceptance of interventions and adherence to prevention measures (38).

Similarly, the impact of social and economic factors on epidemic response has also been addressed. For example, income inequality and crowded living conditions were associated with a higher risk of COVID-19 spread (39). Another study in the United States found that the availability of sick leave and other social protections enhance the ability of individuals to stay home when sick and prevent the spread of a disease (40).

### 3.4. The use of technology and innovation in epidemic response

Technology and innovation can play an important role in epidemic response, from developing new treatments and vaccines to implementing digital tools for surveillance and monitoring (41). The holistic approach to the impact of technology and innovation on epidemic response involves considering the interplay between technological advancements, innovation and epidemic response strategies. This includes exploring the potential of new and emerging technologies such as telemedicine (42), mobile health (43), artificial intelligence (44), and big data analytics (45) to improve disease surveillance, diagnosis and treatment.

The holistic approach also involves examining the impact of technology and innovation on the social, economic and environmental factors that influence epidemic response. For example, the use of technology to facilitate remote work and telecommuting can help reduce the spread of infectious diseases in the workplace, while also mitigating the economic impact of quarantine measures. Additionally, the holistic approach to technology and innovation in epidemic response involves considering the ethical implications of using technology in public health interventions. This includes ensuring that the collection and use of personal health data is carried out in a responsible and transparent manner and that vulnerable population are not further marginalized by technological advancements.

### 3.5. The limitation in resources

Limitations in resources can significantly impact the effectiveness of a holistic approach to epidemic response. In resource-poor settings, the implementation of a holistic approach may be challenging due to a lack of adequate healthcare infrastructure, funding, an efficient and effective supply chain and trained personnel. This can result in inadequate surveillance and response systems, leading to delayed identification and response to outbreaks, and poorer mental and emotional well-being.

A supply chain in an epidemic response refers to the network of organizations, individuals, activities, information and resources involved in the production, transportation, storage and distribution of goods and services that are critical to the response efforts (46). In the context of an epidemic, the supply chain plays a crucial role in ensuring that necessary medical supplies, equipment and other resources are available in the right quantities, at the right time and in the right place to respond to the crisis (47). This includes items such as personal protective equipment (PPE), vaccines, diagnostic tests, medicines and other medical supplies. A well-functioning supply chain is essential to ensure that there is no shortage of critical supplies or delays in their delivery to affected areas (48). The supply chain also plays a critical role in ensuring that the response efforts are sustainable in the long term by maintaining an adequate supply of essential items and preventing stockouts. Effective supply chain management can help to minimize waste, reduce costs and ensure that resources are allocated efficiently to support the response efforts. This can be achieved through the coordination and collaboration among various stakeholders involved in the supply chain, including governments, international organizations, healthcare providers, manufacturers, distributors and logistics companies.

The lack of resources can also impact the implementation of innovative technologies and interventions that can enhance epidemic response. For example, the use of telemedicine and digital health solutions may be limited in resource-poor settings due to poor internet connectivity and inadequate infrastructure. Furthermore, the implementation of sustainable and environmentally friendly interventions may also be challenging due to limited resources and funding.

A holistic approach regarding resources in epidemic response involves considering several factors that yield resource constraints and addressing them in a coordinated and efficient manner. This approach may involve:

1. Prioritization: Prioritizing the most critical resources, such as healthcare personnel, medical supplies and equipment, in order to ensure they are allocated to areas with the greatest need.
2. Resource sharing: Encouraging sharing and collaboration among different organizations and agencies involved in epidemic response to optimize the use of available resources.
3. Innovation: Encouraging innovation in developing new tools, technologies and strategies that can maximize the impact of limited resources in epidemic response.
4. Community involvement: Engaging local communities and stakeholders in the epidemic response to identify local resources that can be leveraged to support the response efforts.
5. Capacity building: Investing in capacity building programs that aim to enhance the skills and knowledge of healthcare personnel, researchers and other stakeholders involved in epidemic response.
6. Advocacy: Advocating for increased funding and resources to support epidemic response efforts, including research and development of new technologies, infrastructure improvements and training programs.

### 3.6. Enhanced support for the mental and emotional well-being of individuals and communities affected by epidemics

This involves addressing public fears and perceptions, as well as promoting the utilization of routine healthcare services regardless of a patient’s status. Emphasizing the importance of seeking regular healthcare services helps ensuring that individuals receive necessary medical attention and maintain their overall well-being during epidemics (19). By allaying fears and encouraging routine healthcare access, the negative effects of fear and stigma associated with the epidemic can be mitigated, promoting a healthier and more resilient community.

**Figure 1:**
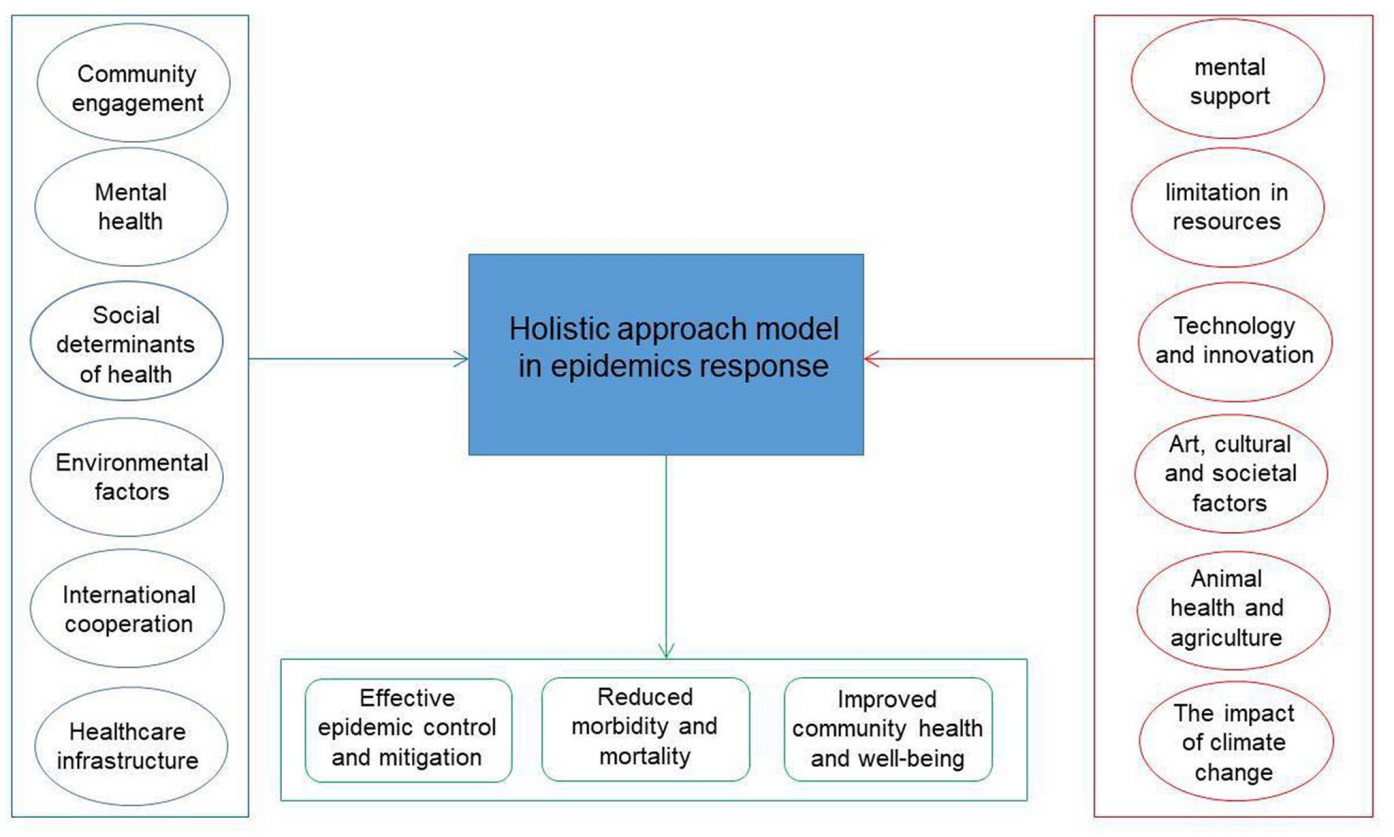
A holistic approach conceptual model in epidemics.

The proposed holistic approach conceptual model in epidemics is illustrated in Figure 1. The inputs are divided in two parts: The ones at the left have already been mentioned in the literature. The ones at the right have not been previously addressed and it is necessary to infuse them in epidemic response. The outputs are the desired results: effective epidemic control, reduced morbidity and mortality and increased community health level.

## 4. Limitations

The implementation of a holistic approach to epidemic response, while conceptually beneficial, can face several challenges that may hinder its effectiveness. It can encounter various challenges that may impede its successful execution. Some potential drawbacks include:

1. Complexity and Coordination: Holistic approaches often involve multiple sectors and stakeholders, requiring intricate coordination and collaboration. The complexity of aligning diverse strategies and actors can lead to delays, conflicts and difficulties in achieving consensus.
2. Resistance to Change: Individuals, communities, or institutions might resist changes that disrupt existing norms or practices, even if the changes are meant to improve overall well-being.
3. Vulnerable and marginalized communities: While holistic approaches intend to address a range of issues, benefits might not be distributed equitably across all segments of the population. Vulnerable and marginalized communities might still experience disparities in accessing the benefits of the approach because they may have limited access to healthcare and information, particularly in low-income countries, hindering their ability to make informed decisions about their health and well-being. Marginalized communities may face challenges in participating in decision-making processes or may not have their voices adequately heard. These communities frequently bear a disproportionate burden of environmental risks, which can result from geographical isolation, financial constraints and cultural disparities.
4. Political Barriers: Bureaucratic hurdles and policies might not be designed to accommodate holistic approaches, leading to legal and bureaucratic barriers that hinder implementation. Moreover, holistic approaches often aim for long-term impact, which might not align with short-term political or funding cycles. This can create challenges in garnering sustained support and commitment from stakeholders.

## 5. Conclusion

The adoption of a holistic approach in epidemics response is crucial for effectively addressing the multifaceted challenges posed by infectious disease outbreaks. This approach encompasses a comprehensive understanding of the epidemic, involving diverse stakeholders and disciplines and integrating various preventive, curative and supportive measures. The approach recognizes the interconnectedness of medical, social, economic and environmental factors and emphasizes the importance of collaboration and coordination among stakeholders from different sectors.

Furthermore, a holistic approach highlights the significance of community engagement, cultural sensitivity and empowerment, ensuring that the response strategies are tailored to the specific needs and contexts of affected communities. It also acknowledges the value of traditional medicine and complementary practices, integrating them into the broader healthcare framework. Finally, it promotes preparedness and resilience, emphasizing the importance of continuous monitoring, evaluation and learning, addressing aspects that have not been fully explored.

In conclusion, the holistic approach yields great potential in enhancing the ability to prevent, control and mitigate the impact of epidemics. By embracing a comprehensive and integrated response strategy, a more resilient, equitable, and effective healthcare system, better equipped to protect the health and well-being of individuals and communities in the face of epidemics, can be fostered.

## Author contributions

IT: Conceptualization, Writing – original draft. NR: Supervision, Writing – review & editing.

## References

[1] Porta M. A dictionary of epidemiology. 6th ed. USA: Oxford University Press (2016).

[2] Jan RW Smuts C. Jan Christiaan Smuts 1870-1950. Society. (1952) 8:271–3. doi: 10.1098/rsbm.1952.0017

[3] Bedford J Farrar J Ihekweazu C Kang G Koopmans M Nkengasong J. A new twenty-first century science for effective epidemic response. Nature. (2019) 575:130–6. doi: 10.1038/s41586-019-1717-y, PMID: 31695207 PMC7095334

[4] Johnson A. Education psychology: Theories of learning and human development. Mankato: National Science Press (2014).

[5] Woods S. Holism in health care: patient as person. handbook of the philosophy of medicine. Dordrecht: Springer (2015).

[6] Callan J. Holistic health or holistic hoax? JAMA. (1979) 241:1156. doi: 10.1001/jama.1979.03290370060033 762771

[7] Yahn G. The impact of holistic medicine, medical groups, and health concepts. JAMA. (1979) 242:2202–5. doi: 10.1001/jama.242.20.2202 490807

[8] Shafran R Bennett S McKenzie S. Interventions to support integrated psychological care and holistic health outcomes in paediatrics. Healthcare. (2017) 5:44. doi: 10.3390/healthcare5030044, PMID: 28812985 PMC5618172

[9] Gordon J. Holistic medicine: advances and shortcomings. West J Med. (1982) 136:546–51. PMID: 7113200 PMC1273970

[10] Filej B Kaucic B. Holistic nursing practice. South East Europ J Public Health. (2013) 3:1–7.

[11] Carnevale J. Employee adjustment and well-being in the era of COVID-19: implications for human resource management. J Bus Res. (2020) 116:183–7. doi: 10.1016/j.jbusres.2020.05.037, PMID: 32501303 PMC7241356

[12] Li J. (2020). International science council. [Online]. Available at: https://council.science/current/blog/a-more-holistic-approach-will-help-avert-pandemics/ [Accessed March 23, 2021].

[13] Patel A Jernigan D. Initial public health response and interim clinical guidance for the 2019 novel coronavirus outbreak. Am J Transplant. (2020) 20:889–95. doi: 10.1111/ajt.15805 32745377 PMC7159597

[14] Ha B Quang N Mirzoev T Tai N Thai P Dinh P. Combating the COVID-19 epidemic: experiences from Vietnam. Int J Environ Res Public Health. (2020) 17:3125. doi: 10.3390/ijerph17093125 32365851 PMC7246912

[15] Lee D Heo K Seo Y. COVID-19 in South Korea: lessons for developing countries. World Dev. (2020) 135:105057. doi: 10.1016/j.worlddev.2020.105057 32834374 PMC7321039

[16] Abramowitz S Bedson J. Community engagement in disease outbreak preparedness and response: Lessons from recent outbreaks, key concepts, and quality standards for practice. Cham: Springer (2022).

[17] UNICEF (2018). www.unicef.org. [Online]. Available at: https://www.unicef.org/media/52171/file [Accessed April 12, 2023].

[18] UN Volunteers (2020). The importance of mental health during the COVID-19 pandemic. Available at: https://www.unv.org/Success-stories/importance-mental-health-during-covid-19-pandemic. [Accessed July 10, 2023].

[19] WHO (2020). WHO [Online]. Available at: https://www.who.int/southeastasia/news/detail/06-08-2020-maintain-essential-health-services-during-covid-19-response-who [Accessed February 22, 2022].

[20] CDC (2022). Available at: https://www.cdc.gov/about/sdoh/addressing-sdoh.html [Accessed April 11, 2023].

[21] WHO (2008). Available at: https://www.who.int/publications/i/item/9789241563703 [Accessed April 11, 2023].

[22] Valsamatzi-Panagiotou A Penchovsky R. Environmental factors influencing the transmission of the coronavirus 2019: a review. Environ Chem Lett. (2022) 20:1603–10. doi: 10.1007/s10311-022-01418-9, PMID: 35221835 PMC8859930

[23] Katz R Wentworth M Quick J Arabasadi A Harris E Geddes K. Enhancing public–private cooperation in epidemic preparedness and response. World Med Health Policy. (2018) 10:420–5. doi: 10.1002/wmh3.281

[24] Kokudo N Sugiyama H. Call for international cooperation and collaboration to effectively tackle the COVID-19 pandemic. Glob. Health Med. (2020) 2:60–2. doi: 10.35772/ghm.2020.01019 PMC7731425 33330778

[25] WHO (2014). Available at: https://apps.who.int/iris/bitstream/handle/10665/151281/9789241548939_eng.pdf [Accessed April 11, 2023].

[26] International Peace Institute (2017). Global pandemics and global public health. Report. International Peace Institute. p. 16–20.

[27] Mora C McKenzie T Gaw IM Dean J Hammerstein H Knudson T. Over half of known human pathogenic diseases can be aggravated by climate change. Nat Clim Chang. (2022) 12:869–75. doi: 10.1038/s41558-022-01426-1 35968032 PMC9362357

[28] Cook RA Karesh WB Osofsky SA. (2004). One world, one health: Building interdisciplinary bridges to health in a globalized world. [Online]. New York; Available at: http://www.oneworldonehealth.org/sept2004/owoh_sept04.html. [Accessed July 10, 2023].

[29] Petrovan S Aldridge D Bartlett H Bladon A Booth H Broad S. Post COVID-19: a solution scan of options for preventing future zoonotic epidemics. Biol Rev. (2021) 96:2694–715. doi: 10.1111/brv.12774, PMID: 34231315 PMC8444924

[30] Ferri M Lloyd-Evans M. The contribution of veterinary public health to the management of the COVID-19 pandemic from a one health perspective. One Health. (2021) 12:100230. doi: 10.1016/j.onehlt.2021.100230, PMID: 33681446 PMC7912361

[31] Miller L Gee P Katz R. The importance of understanding COVID-19: the role of knowledge in promoting adherence to protective behaviors. Front Public Health. (2021) 9:1–8. doi: 10.3389/fpubh.2021.581497, PMID: 33889557 PMC8055953

[32] Bayeh R Yampolsky M Ryder A. The social lives of infectious diseases: why culture matters to COVID-19. Front Psychol. (2021) 12:648086. doi: 10.3389/fpsyg.2021.648086, PMID: 34630195 PMC8495420

[33] WHO (2022). WHO.int. Available at: https://www.who.int/docs/default-source/coronaviruse/covid19-stigma-guide.pdf [Accessed February 22, 2022].

[34] WHO (2022). Who.int. Available at: Available at: https://www.who.int/health-topics/health-financing#tab=tab_1 [Accessed February 11, 2023].

[35] Epstein R Bluethenthal A Visser D Pinsky C Minkler M. Leveraging arts for justice, equity, and public health: the Skywatchers program and its implications for community-based health promotion practice and research. Health Promot Pract. (2021) 22:91S–100S. doi: 10.1177/1524839921996066 33942636

[36] Manguvo A Mafuvadze B. The impact of traditional and religious practices on the spread of Ebola in West Africa: time for a strategic shift. Pan Afr Med J. (2015) 22:9. doi: 10.11604/pamj.supp.2015.22.1.6190 PMC4709130 26779300

[37] Jalloh M Robinson S Corker J Li W Irwin K Barry A. Knowledge, attitudes, and practices related to Ebola virus disease at the end of a National Epidemic-Guinea. Morb Mort Weekly Rep. (2017) 66:1109–15. doi: 10.15585/mmwr.mm6641a4 PMC5689093 29049279

[38] Merten S Schaetti C Manianga C Lapika B Chaignat CL Hutubessy R. Local perceptions of cholera and anticipated vaccine acceptance in Katanga province, Democratic Republic of Congo. BMC Public Health. (2013) 13:60. doi: 10.1186/1471-2458-13-60 23339647 PMC3626893

[39] Wachtler B Michalski N Nowossadeck E Diercke M Wahrendorf M Santos-Hövener C. Socioeconomic inequalities and COVID-19- a review of the current international literature. J Health Monit. (2020) 9:3–17. doi: 10.25646/7059 PMC8734114 35146298

[40] Kim D. Paid sick leave and risks of all-cause and cause-specific mortality among adult workers in the USA. Int J Environ Res Public Health. (2017) 14:1247. doi: 10.3390/ijerph14101247, PMID: 29048337 PMC5664748

[41] OECD (2021). Available at: https://www.oecd.org/coronavirus/policy-responses/how-will-covid-19-reshape-science-technology-and-innovation-2332334d/ [Accessed April 11, 2023]

[42] Dasgupta A Deb S. Telemedicine: a new horizon in public health in India. Indian. J Commun Med. (2008) 33:3–8. doi: 10.4103/0970-0218.39234 PMC2782224 19966987

[43] Istepanian R. Mobile health (m-health) in retrospect: the known unknowns. Int J Environ Res Public Health. (2022) 19:3747. doi: 10.3390/ijerph19073747, PMID: 35409431 PMC8998037

[44] Davenport T Kalakota R. The potential for artificial intelligence in healthcare. Future Healthc J. (2019) 6:94–8. doi: 10.7861/futurehosp.6-2-94, PMID: 31363513 PMC6616181

[45] Wang L Cheryl A. Big data analytics in healthcare systems. Int J Math Eng Manag Sci. (2019) 4:17–26. doi: 10.33889/IJMEMS.2019.4.1-002

[46] Behdad E Karimi H Bakhshi A Aghsami A Rabbani M. Designing humanitarian logistics network for managing epidemic outbreaks in disasters using internet-of-things. A case study: An earthquake in Salas-e-Babajani city. Comput Ind Eng. (2023) 175:108821. doi: 10.1016/j.cie.2022.108821, PMID: 36506844 PMC9720066

[47] Dasaklis T Pappis C Rachaniotis N. Epidemics control and logistics operations: a review. Int J Prod Econ. (2012) 139:393–410. doi: 10.1016/j.ijpe.2012.05.023

[48] Waters D. Supply chain risk management: Vulnerability and resilience in logistics. London and Philadelphia: Kogan Page Limited (2007).

